# Metagenomic next-generation sequencing for pulmonary infections diagnosis in patients with diabetes

**DOI:** 10.1186/s12890-023-02441-4

**Published:** 2023-04-28

**Authors:** Siqin Zhang, Jing Ou, Yuxue Tan, Bin Yang, Yaoyao Wu, Lin Liu

**Affiliations:** 1 Department of Endocrinology and Metabolism, Guizhou Provincial People’s Hospital, Guiyang, Guizhou, 550002 China; 2 School of Medicine, Zunyi Medical University, Zunyi, Guizhou, 563000 China; 3 Department of Central Laboratory, Guizhou Provincial People’s Hospital, Guiyang, Guizhou, 550002 China; 4 Department of Respiratory and Critical Medicine, Guizhou Provincial People’s Hospital, No. 83, Zhongshan East Road, Guiyang, Guizhou, 550002 China; 5 NHC Key Laboratory of Pulmonary Immunological Diseases (Guizhou Provincial People’s Hospital), Guiyang, Guizhou, 550002 China

**Keywords:** Metagenomic next-generation sequencing, Pulmonary infection, Diabetes, Diagnosis

## Abstract

**Background:**

Diabetes mellitus is a major cause of high mortality and poor prognosis in patients with pulmonary infections. However, limited data on the application of metagenomic next-generation sequencing (mNGS) are available for diabetic patients. This study aimed to evaluate the diagnostic performance of mNGS in diabetic patients with pulmonary infections.

**Methods:**

We retrospectively reviewed 184 hospitalized patients with pulmonary infections at Guizhou Provincial People’s Hospital between January 2020 to October 2021. All patients were subjected to both mNGS analysis of bronchoalveolar lavage fluid (BALF) and conventional testing. Positive rate by mNGS and the consistency between mNGS and conventional testing results were evaluated for diabetic and non-diabetic patients.

**Results:**

A total of 184 patients with pulmonary infections were enrolled, including 43 diabetic patients and 141 non-diabetic patients. For diabetic patients, the microbial positive rate by mNGS was significantly higher than that detected by conventional testing methods, primarily driven by bacterial detection (microbes: 95.3% vs. 67.4%, P = 0.001; bacteria: 72.1% vs. 37.2%, P = 0.001). mNGS and traditional tests had similar positive rates with regard to fungal and viral detection in diabetic patients. *Klebsiella pneumoniae* was the most common pathogen identified by mNGS in patients with diabetes. Moreover, mNGS identified pathogens in 92.9% (13/14) of diabetic patients who were reported negative by conventional testing. No significant difference was found in the consistency of the two tests between diabetic and non-diabetic groups.

**Conclusions:**

mNGS is superior to conventional microbiological tests for bacterial detection in diabetic patients with pulmonary infections. mNGS is a valuable tool for etiological diagnosis of pulmonary infections in diabetic patients.

## Introduction

Diabetes mellitus (DM), one of the most common chronic diseases worldwide, is a major cause of high mortality and poor prognosis from pulmonary infections [1–4]. Immune dysfunction, including impaired leukocyte adhesion, chemotaxis and phagocytosis, and depressed bactericidal activity may underlie poor outcomes in diabetic patients [5, 6]. Therefore, pathogen diagnosis should be carried out as soon as possible for diabetic patients with pulmonary infections. However, traditional testing methods have several limitations that make them insufficient for clinical needs, including a low positive culture rate, especially for fastidious microbes, and a restricted pathogen detection spectrum for polymerase chain reaction (PCR) tests [7, 8]. Failure to identify pathogens may lead to the use of unnecessary broad-spectrum antibiotics, exacerbating antibiotic resistance and resulting in both higher costs and poor prognosis.

Metagenomic next-generation sequencing (mNGS) is a novel and promising pathogen detection method that uses high-throughput sequencing and bioinformatics analysis to detect non-targeted pathogens, including bacteria, fungi, viruses and parasites [9, 10]. Recent studies have shown that mNGS is superior to traditional testing methods with regard to the sensitivity and accuracy of pathogen diagnosis [11]. Moreover, Miao et al. reported that the mNGS method was less affected by prior antibiotic treatment compared to conventional tests [12]. Early diagnosis of pathogens in cases of pulmonary infection promotes selection of the optimal antibiotic therapy, shortens the duration of mechanical ventilation, and reduces the mortality rate [13]. Nevertheless, few studies evaluated the diagnostic performance of mNGS in diabetes.

In the present study, we evaluated the diagnostic performance of mNGS in diabetic patients and compared clinical management and outcomes between diabetic and non-diabetic groups. Our findings support the clinical application of mNGS in diabetic patients with pulmonary infections.

## Subjects and methods

### Study participants and design

**Fig. 1:**
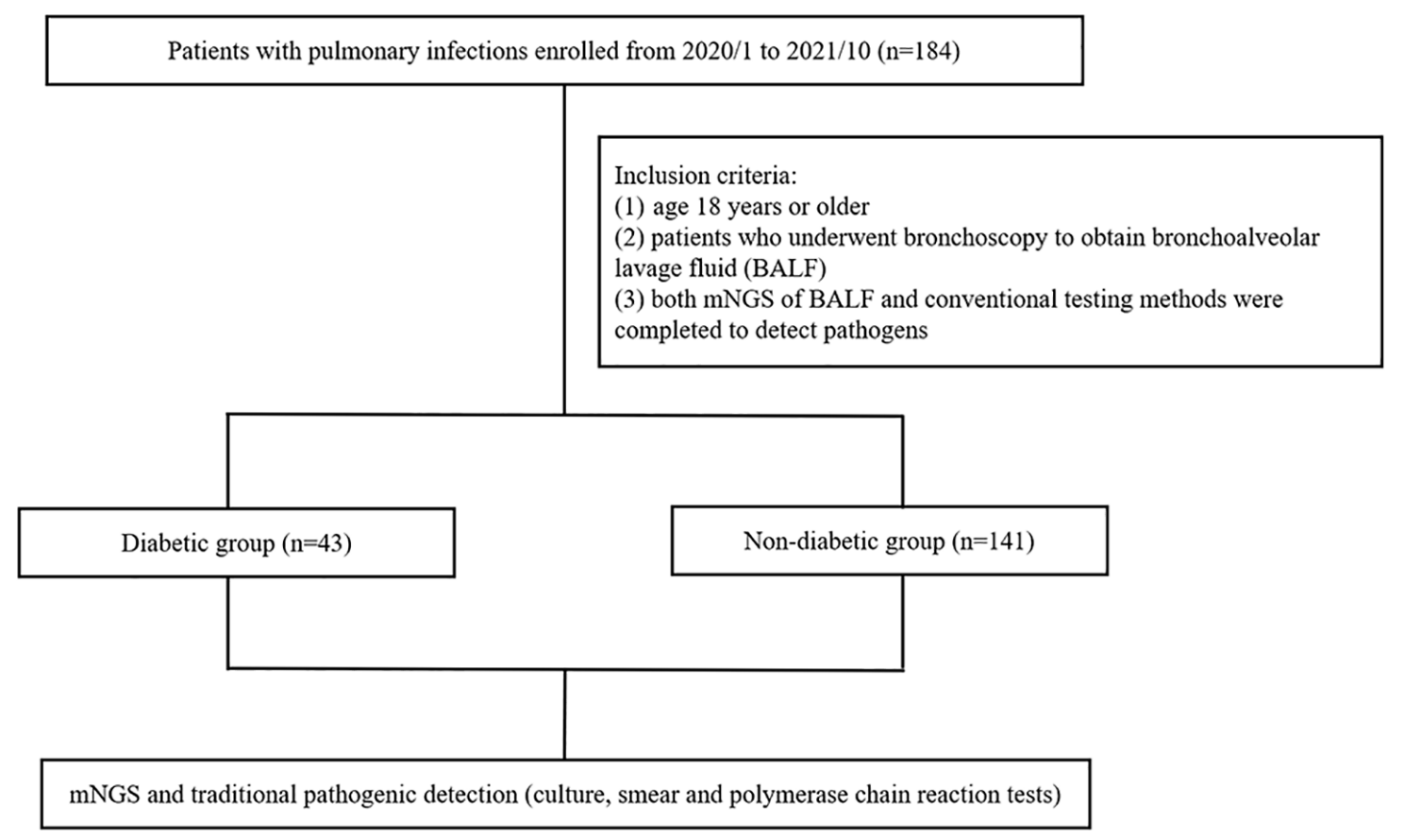
Flow chart of patients screening

We retrospectively reviewed 184 hospitalized patients who presented with pulmonary infections at Guizhou Provincial People’s Hospital between January 2020 to October 2021 (Fig. 1). Inclusion criteria were as follows: (1) age 18 years or older; (2) patients who underwent bronchoscopy to obtain bronchoalveolar lavage fluid (BALF); (3) both mNGS of BALF and conventional testing methods were completed to detect pathogens. Patients were excluded if they met any of the following criteria: (1) patients who refused bronchoscopy or mNGS examination; (2) BALF samples failed to pass quality control for mNGS; (3) incomplete clinical records. Demographic, clinical variables, comorbidities, immunosuppressive state, length of hospital stay, cost, treatment process, and prognosis data were collected for further analysis. This study was approved by the Ethics Committee of Guizhou Provincial People’s Hospital (No. [2020] 500). Informed consent was signed for bronchoscopy and mNGS examination.

Patients were divided into diabetic and non-diabetic groups based on diabetes status. The positive rate by mNGS testing and the consistency between mNGS and conventional testing methods were evaluated in diabetic and non-diabetic patients. Clinical management and outcomes were also compared between diabetic and non-diabetic groups. In this study, conventional tests included bacterial or fungal culture, smear for *Mycobacterium tuberculosis*, and PCR nucleic acids test for *Human gammaherpesvirus 4* (EBV), *Human betaherpesvirus 5* (CMV), *Mycoplasma pneumoniae*, *Mycobacterium tuberculosis*, and *influenza A/B*. Conventional testing specimens included BALF, sputum and blood.

Immunosuppression was defined by the presence of any of the following conditions: chemotherapy or neutropenia during the past month, corticosteroid treatment of more than two weeks, immunosuppressive therapy, human immunodeficiency virus infection, and active tuberculosis [14].

### Sample processing and sequencing

Bronchoscopy was performed by experienced bronchoscopists during hospitalization. For patients with mechanical ventilation, the median time for BALF collection was 1 day after intubation. For patients without mechanical ventilation, the median time for BALF collection was 5 days after admission. Samples were usually taken during acute exacerbations or poor treatment response. BALF was collected and transported according to standard procedures. First, 0.5 ml BALF sample and 1 g of 0.5 mm glass beads were loaded into a 1.5 ml microcentrifuge tube, which was attached to a horizontal platform of a vortex stirrer and agitated vigorously at 2800–3200 RPM for 30 min. Subsequently, 0.3 ml sample was isolated, and DNA was extracted using the TIANamp Micro DNA Kit (DP316, Tiangen Biotech).

DNA libraries were constructed via DNA-fragmentation, terminal repair, phosphorylation, adapter-ligation and PCR amplification. DNA libraries were qualified by Agilent 2100 Bioanalyzer and Qubit dsDNA HS Assay Kit (Thermo Fisher Scientific Inc.) [15]. High-quality DNA libraries were subjected to denaturation and circularization to generate single-stranded circular structures. The circularized DNA library was then transformed into DNA nanoballs (DNBs) via rolling circle replication (RCA). Prepared DNBs were loaded onto the sequencing chip and sequenced using the BGISEQ-50 platform.

### Bioinformatic analyses

High-quality sequencing data was obtained by removing low-quality and short (length < 35 bp) sequences before further analyses. Removing low-quality sequences were qualified by in-house software stat_split_bc2 and get_umhost_IC_qc. The quality of checking duplicate sequences were controlled by samtools and prinseq_lite.pl [16]. Human sequence data were excluded from high-quality sequences by mapping to the human reference genome (hg19) using Burrows-Wheeler Alignment. Finally, remaining sequence data were identified and classified as derived from bacteria, viruses, fungi or parasites by alignment to microbial genome databases using Burrows-Wheeler Alignment [17]. The microbial genome databases were downloaded from refseq and genbank in the National Center for Biotechnology Information (ftp://ftp.ncbi.nlm.nih.gov/genomes/). The databases contained 2,473 bacterial species, 4,061 viral species, 199 fungal species, and 135 parasites related to human diseases.

### Threshold criteria for mNGS result interpretation

For different types of microorganisms, the criteria for a positive mNGS test were defined as follows: [18] (1) the stringently mapped read number (SMRN) for bacteria, virus, fungus, mycoplasma or chlamydia was no less than 3; (2) at least one unique read was aligned to the *Mycobacterium tuberculosis* genome; (3) positive parasite detection was considered if the SMRN was no less than 100.

Suspected background microorganisms were excluded from mNGS test reports. Pathogens found in pulmonary infection were identified by two experienced clinicians based on clinical features, imaging, and treatment response.

### Statistical analysis

Continuous variables (normal distribution) were expressed as mean ± standard deviation, and continuous variables with nonnormal distribution were described as median and interquartile range (IQR) values. Categorical variables were described as numbers and percentages. Continuous data for the two groups were analyzed by t test or Mann-Whitney test. Analyses of categorical variables were performed by chi-square test. P values < 0.05 were considered statistically significant. All statistical analyses were conducted using Statistical Product and Service Solution (SPSS) 22.0 (IBM, USA).

## Results

### Demographic characteristics and clinical outcomes

A total of 184 patients hospitalized with pulmonary infections were enrolled in this study. Demographic and clinical characteristics of the patients are shown in Table 1. The mean age of the patients was 62.02 ± 14.41 years, and 119 (64.7%) were men. Among this cohort, 139 (75.5%) patients had underlying diseases, including 43 (23.4%) cases of diabetes, 70 (38.0%) cases of hypertension, 20 (10.9%) cases of coronary heart disease, 13 (7.1%) cases of cerebral infarction, 31 (16.8%) cases of cancer, 74 (40.2%) cases of renal insufficiency, and 41 (22.3%) cases of immunosuppression. The median number of total sequence reads of BALF samples from 184 patients was 42,477,808 (30,373,771–69,173,794).

**Table 1:** Demographic characteristics and clinical outcomes of patients with pulmonary infections

	Diabetic group (n = 43)	Non-diabetic group (n = 141)	P value
Age, (years)	63.4 ± 13.6	61.6 ± 14.7	0.477
Sex, male n (%)	30 (69.8%)	89 (63.1%)	0.425
Community-acquired pneumonia, n (%)	42 (97.7%)	135 (95.7%)	0.902
Comorbidities, n (%)			
Hypertension	23 (53.5%)	47 (33.3%)	0.017
Coronary heart disease	8 (18.6%)	12 (8.5%)	0.114
Cerebral infarction	7 (16.3%)	6 (4.3%)	0.019
Cancer	5 (11.6%)	26 (18.4%)	0.296
Renal insufficiency	20 (46.5%)	54 (38.3%)	0.336
Immunosuppression	8 (18.6%)	33 (23.4%)	0.508
Duration of mechanical ventilation(hours)	0 (0-239.3)	0 (0-101.0)	0.272
Mechanical ventilation ratio	19 (44.2%)	54 (38.3%)	0.490
Length of stay (days)			
In hospital	16.0 (12.0–24.0)	15.0 (10.0–22.0)	0.341
In ICU	6.0 (0–13.0)	0 (0–9.0)	0.024
Medical costs (10,000 RMB)	5.9 (2.5–17.0)	3.5 (1.7–8.6)	0.028
Remission rate	28 (65.1%)	95 (67.4%)	0.783
In-hospital mortality rate	3 (7.0%)	9 (6.4%)	1.000

Abbreviations: ICU, intensive care unit

All eligible patients were divided into diabetic (43 cases) and non-diabetic groups (141 cases). There were no significant differences in age or gender distribution between the two groups. The mean random blood glucose of patients in the diabetic group was 12.20 ± 7.02 mmol/L, and the mean duration of diabetes was 5.96 ± 5.71 years. Of the 43 patients with diabetes, 38 (88.4%) cases had type 2 diabetes and 5 (11.6%) had undetermined diabetes types. 30 (69.8%) patients were treated with insulin. Compared to the non-diabetic group, diabetic patients had more chronic comorbidities, especially hypertension and cerebral infarction (all P < 0.05). No significant difference was found in the proportion of community-acquired pneumonia between the two groups. The prevalence of co-infection was 61.9% in the diabetic group and 66.4% in the non-diabetic group. There was no significant difference in the prevalence of co-infection between the two groups.

In terms of clinical outcomes, patients with diabetes had significantly longer intensive care unit (ICU) stays (P = 0.024) and higher medical costs (P = 0.028) compared to non-diabetic patients. However, no significant differences were observed in mechanical ventilation duration, in-hospital mortality or remission rate between the two groups (Table 1).

### Diagnostic performance of mNGS in diabetic and non-diabetic patients

**Fig. 2:**
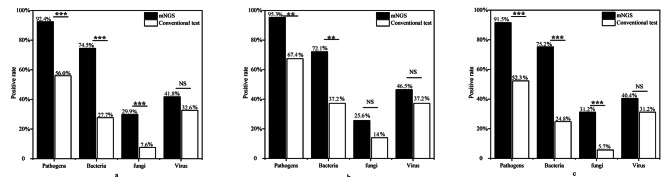
Diagnostic performance of mNGS assay for pathogen detection. (**a**) Rates of pathogen detection by mNGS and conventional testing in all patients. (**b**) Rates of pathogen detection by mNGS and conventional testing in diabetic patients. (**c**) Rates of pathogen detection by mNGS and conventional testing in non-diabetic patients. *P < 0.05, **P < 0.01 and ***P < 0.001; NS not significant

In a total of 184 patients, the microbial positive rate for mNGS was significantly higher than that for conventional testing (92.4% vs. 56.0%, P < 0.001). For bacterial and fungal detection, mNGS had higher percentages of positive samples than conventional testing methods (bacteria: 74.5% vs. 27.7%, P < 0.001; fungi: 29.9% vs. 7.6%, P < 0.001). The positive rates of the two methods were similar with regard to viral detection (Fig. 2a).

In diabetic patients, the microbial positive rate was 95.3% (41 of 43 patients) for mNGS versus 67.4% (29 of 43 patients) by conventional testing methods (P = 0.001). The percentages of mNGS-positive samples were significantly higher than those of conventional test-positive samples with regard to bacterial detection (72.1% vs. 37.2%, P = 0.001). However, there were no significant differences in positivity rates between mNGS and traditional testing with regard to fungal or viral detection (Fig. 2b).

In non-diabetic patients, significant differences were detected in the positivity rates of microbial detection between mNGS and conventional testing (91.5% vs. 52.3%, P < 0.001). Moreover, mNGS had higher positive detection rates for bacteria and fungi than conventional tests (all P < 0.001). For viral detection, the positivity rates of the two methods were similar (Fig. 2c).

### Pathogens detected by mNGS and conventional testing

#### Identification of pathogens in diabetic patients by mNGS

**Fig. 3:**
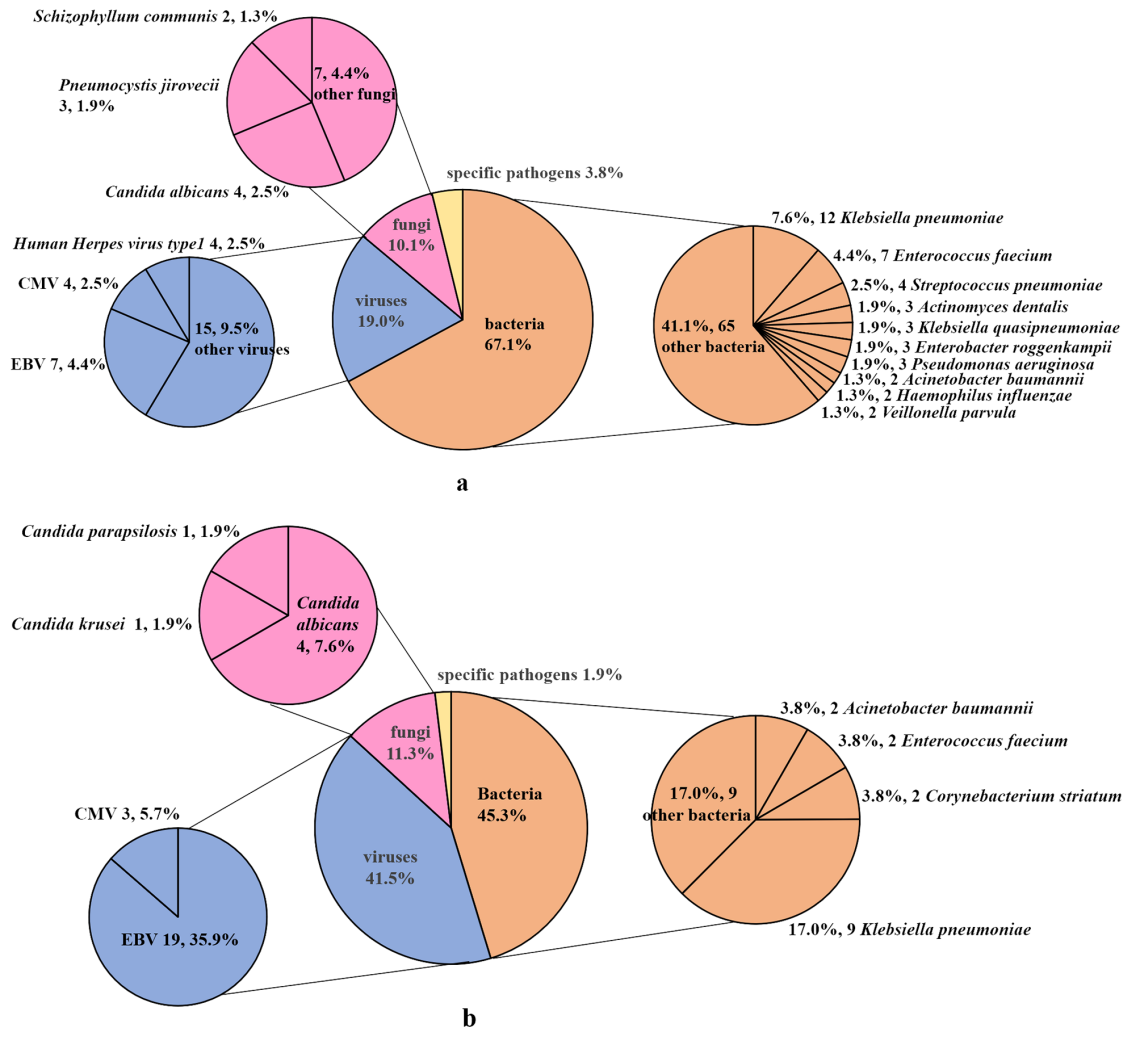
Numbers of organisms identified by mNGS and conventional testing in diabetic patients. (**a**) Pathogens detected by mNGS in diabetic patients. (**b**) Pathogens detected by conventional testing in diabetic patients. EBV, Human gammaherpesvirus 4; CMV, Human betaherpesvirus 5

A total of 158 pathogens were detected by mNGS in 43 patients with diabetes. The median number of sequence reads were 49 (12–298). These included 106 bacteria, 16 fungi, 30 viruses and 6 specific pathogens, which were identified as two *Mycobacterium tuberculosis*, two *chlamydia* and two *mycoplasmas*. Figure 3a lists the distribution of bacteria, fungi and viruses identified by mNGS in diabetic patients. *Klebsiella pneumoniae* (12/158, 7.6%), *Enterococcus faecium* (7/158, 4.4%) and *Streptococcus pneumoniae* (4/158, 2.5%) were the most common bacteria. The median number of sequence reads for each of the three bacteria were as follows: *Klebsiella pneumoniae* 457 (20-4413), *Enterococcus faecium* 495 (86-5913), and *Streptococcus pneumoniae* 35 (4-128). The most frequently detected fungi were *Candida albicans* (4/158, 2.5%) and *Pneumocystis jirovecii* (3/158, 1.9%). And the median number of sequence reads for each of the two fungi were as follows: *Candida albicans* 12 (3-754) and *Pneumocystis jirovecii* 464 (3-2341). In addition, the most commonly detected viruses were EBV (7/158, 4.4%) and CMV (4/158, 2.5%). The median number of sequence reads for each of the two viruses were as follows: EBV 7 (5–12) and CMV 56 (15–121).

#### Identification of pathogens in diabetic patients by conventional tests

A total of 53 pathogens were identified by conventional tests in diabetic patients. The pathogens included 24 bacteria, 6 fungi, 22 viruses and one specific pathogen, which was identified as *Mycobacterium tuberculosis*. All detected pathogens are listed in Fig. 3b. Among the microbes isolated, *Klebsiella pneumoniae* (9/53, 17.0%), *Acinetobacter baumannii* (2/53, 3.8%), *Enterococcus faecalis* (2/53, 3.8%) and *Corynebacterium striatum* (2/53, 3.8%) were the most frequently detected bacteria. The most commonly detected fungus was *Candida albicans* (4/53, 7.6%). Moreover, a total of 16 patients were virus-positive, and the identified viruses were EBV (19/53, 35.9%) and CMV (3/53, 5.7%).

#### Identification of Pathogens in patients with negative conventional tests by mNGS

Of the 14 diabetic patients who had negative results by conventional tests, pathogen genome was detected in 13 cases. A total of 24 pathogens were detected by mNGS in these patients. The most commonly detected bacteria were *Klebsiella pneumoniae* (2/24, 8.3%) and *Enterococcus faecalis* (2/24, 8.3%). The fungus and virus detected by mNGS were *Aspergillus* (1/24, 4.2%) and CMV (1/24, 4.2%), respectively, in diabetic patients with negative conventional testing results.

Of the 66 non-diabetic patients who were negative for traditional methods, 59 tested positive for mNGS. A total of 100 pathogens were identified by mNGS in these patients. *Abiotrophia defectiva* (5/100, 5.0%) and *Streptococcus pneumoniae* (5/100, 5.0%) were the most frequently detected bacteria. The most commonly detected fungus was *Aspergillus fumigatus* (6/100, 6.0%) and the most common virus was CMV (6/100, 6.0%).

### Concordance between mNGS and conventional tests

**Fig. 4:**
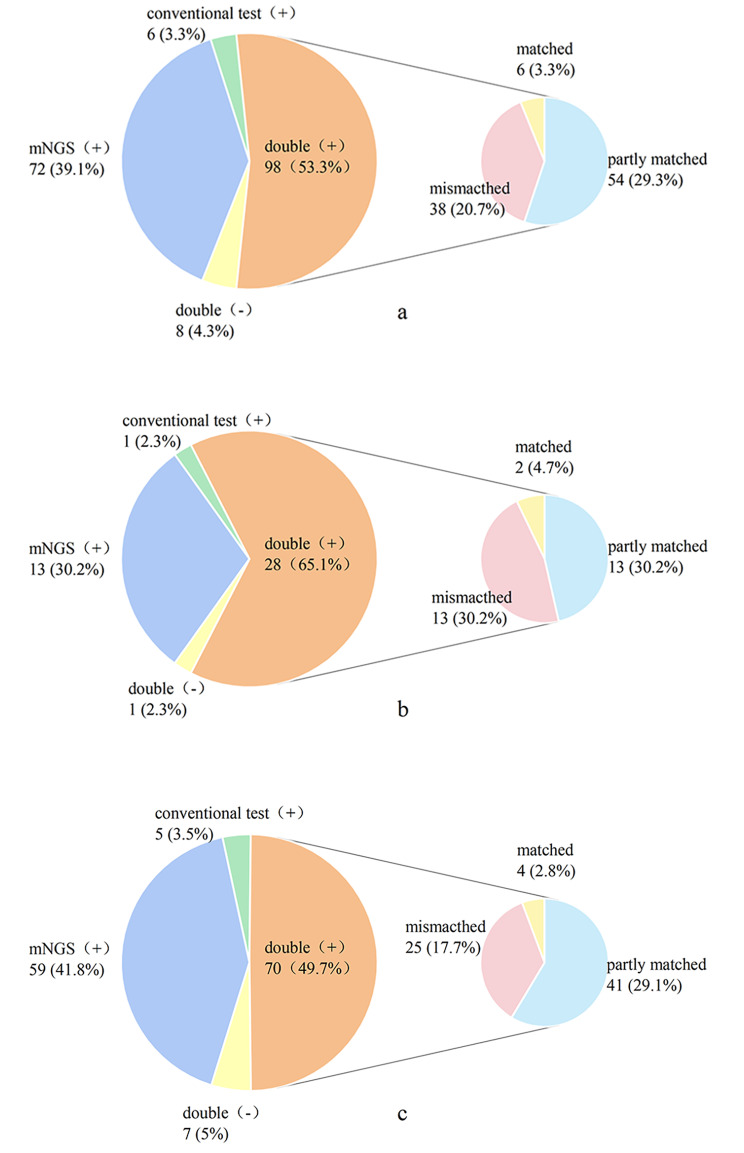
Concordance between mNGS and conventional testing in pathogen detection. (**a**) Concordance between mNGS and conventional testing in all patients. (**b**) Concordance between mNGS and conventional testing in diabetic patients. (**c**) Concordance between mNGS and conventional testing in non-diabetic patients

Of the 184 patients with pulmonary infections, 98 (53.3%) had positive results by both mNGS and conventional testing. In these 98 cases, the consistency between mNGS and conventional tests were as follows: (1) totally matched in 6 (3.3%) cases, (2) partially matched in 54 (29.3%) cases, (3) completely mismatched in 38 (20.7%) cases (Fig. 4a).

Among the 43 diabetic patients, 28 patients (65.1%) were positive by both mNGS and conventional testing, and one patient (2.3%) was negative by both detection methods. Meanwhile, a total of 13 (30.2%) cases were positive by mNGS only, and one case (2.3%) was positive by conventional tests only. Moreover, mNGS identified pathogens in 92.9% (13/14) of patients who were tested negative by conventional test. For the 28 cases that tested positive by both methods, the results of mNGS and conventional tests were totally matched in two (4.7%) cases, completely mismatched in 13 (30.2%) cases and partially matched in 13 (30.2%) cases (Fig. 4b).

Of the 141 non-diabetic patients, 70 (49.7%) had positive results by both mNGS and conventional testing. A total of 59 (41.8%) cases were positive by mNGS detection and negative by conventional testing methods. Five (3.5%) cases were considered positive by conventional testing methods only. In addition, 7 (5.0%) cases were negative by both tests. Of the 70 cases that tested positive by both methods, the results of mNGS and conventional testing methods were totally consistent in 4 (2.8%) cases and completely inconsistent in 25 (17.7%) cases. The remaining 41 (29.1%) cases had partially consistent results (Fig. 4c).

A comparison of the concordance between mNGS and conventional testing in diabetic and non-diabetic patients is shown in Table 2. There was no significant difference in the consistency of pathogen detection results between diabetic and non-diabetic groups for cases defined as positive by both methods (P = 0.550).

**Table 2:** Concordance of mNGS and conventional testing between diabetic and non-diabetic groups for double-positive cases

	Diabetic group (n = 28)	Non-diabetic group (n = 70)	P value
completely matched	2 (7.1%)	4 (5.7%)	0.550
partly matched	13 (46.4%)	41 (58.6%)	
mismatched	13 (46.4%)	25 (35.7%)	

## Discussion

This is a retrospective study on the diagnostic performance of mNGS for pulmonary infections in diabetic patients. In diabetic patients, the rate of positive microorganism detection by mNGS was significantly higher than that by conventional testing methods, especially with regard to bacterial detection. mNGS was positive in 92.9% of diabetic patients who were reported negative by conventional testing. No significant differences were found in the consistency of the two tests between diabetic and non-diabetic groups.

In diabetic patients, we observed that the positive rate of mNGS for pathogens in cases of pulmonary infection was significantly higher than that of conventional tests, mainly driven by superior bacterial detection (microbes: 95.3% vs. 67.4%, P = 0.001; bacteria: 72.1% vs. 37.2%, P = 0.001). Consistent with our study, Fang et al. [14] showed that pathogens could be identified in a significantly higher proportion of patients using mNGS compared to culture. Another study also reported that mNGS outperformed traditional tests with regard to bacterial detection [18]. Unlike the aforementioned studies, Miao et al. [12] reported no significant difference between mNGS and conventional tests for bacterial detection. This discrepancy may arise from differences in sample type or disease severity. These results suggest that mNGS improves the etiological detection rate of pulmonary infection for the diabetic population, mainly in bacterial cases.

In this study, *Klebsiella pneumoniae* was the most common pathogen identified by mNGS in diabetic patients. Inconsistent with our results, a recent study reported *Acinetobacter baumannii* as the most frequently detected pathogen in pulmonary infections [14]. This may be explained by the fact that all enrolled patients in that study had ventilator-associated pneumonia. Based on the results of our study, clinicians can provide empirical anti-infective treatment for diabetic patients with pulmonary infections who have not yet obtained etiological information.

In terms of consistency between mNGS and conventional testing results, we found that mNGS identified pathogens in 92.9% (13/14) of diabetic patients who were negative by conventional testing. Nearly a third of cases in the diabetic group were considered positive by mNGS detection only. These causative pathogens were not detected by conventional tests and might not otherwise be considered by clinicians. Another study observed that 67 of 140 cases (47.9%) tested positive for mNGS while testing negative by traditional methods [18]. These results suggest that mNGS allows the unbiased and untargeted detection of a broad range of potential infectious pathogens, one of the approach’s most attractive advantages.

We observed that the results of the two detection methods were completely mismatched in 46.4% of patients who tested positive for both tests in the diabetic group. Similarly, conflicts between testing results have been reported in previous studies [14, 19]. These inconsistent pathogen results may confuse clinical therapy. One of the possible reasons for this phenomenon is that some special pathogens require strict culture conditions. If clinicians do not consider the infection of these pathogens, it is difficult to detect pathogens by conventional culture methods. We found that *Abiotrophia defectiva* was the predominant mNGS-detected pathogen in patients with negative conventional tests. This is because culturing of *Abiotrophia defectiva* is time-consuming with a low positivity rate. Similarly, previous studies have shown that mNGS can identify difficult-to-culture or rare pathogens, such as *Leptospira* from cerebrospinal fluid, [20] *dengue virus 1* and *Ebola virus* from plasma, [21] and *Aspergillus* and *Corynebacterium striatum* from BALF [14, 19]. An important advantage of mNGS is its ability to detect pathogens that are difficult to identify with traditional testing methods [22]. In addition, contamination of samples in the process of BALF collection or detection may also be one of the reasons for the inconsistent results of the two testing methods. Therefore, when the results of these two methods are inconsistent, clinicians should make a comprehensive judgment based on patient symptoms and imaging or collect additional samples for testing verification.

In the present study, diabetic patients with pulmonary infections had significantly higher costs and longer ICU stays compared to non-diabetic patients. While differences in mortality and remission rates were not statistically significant between the two groups, this may be related to the small sample size. Recent work showed that mNGS can significantly shorten ICU stay length and reduce treatment cost for immunosuppressed patient [23]. Therefore, from an economic perspective, diabetic patients are better candidates for the use of mNGS to assist early diagnosis and targeted therapy, reducing cost and hospital stay length.

This study has several limitations. First, the enrolled patients were subjected to DNA sequencing without RNA sequencing, potentially leading to false negative results. Second, pre-onset blood glucose management and glycosylated hemoglobin levels were not recorded, leading to poor assessment of pre-onset blood glucose control. In addition, drug resistance testing was not performed in this study. Furthermore, the sample size is limited in our study, especially with regard to diabetic patients. Therefore, the diagnostic efficacy of mNGS in patients with diabetes requires further verification in large-scale studies. For more rapid etiological diagnosis of pulmonary infection, real-time metagenomics sequencing can be used in further studies to detect pathogens within a few hours, as well as bacterial analysis and genotyping [24].

## Conclusions

In summary, mNGS is superior to conventional microbiological testing for the detection of bacteria in diabetic patients with pulmonary infections. mNGS has additional advantages in detecting difficult-to-culture and rare pathogens. Moreover, diabetic patients have longer ICU stays and higher costs of pulmonary infection therapy. Therefore, early application of mNGS for etiological diagnosis in diabetic patients may help clinicians make timely treatment decisions, reduce costs, and improve prognosis.

## Data Availability

The datasets generated and/or analyzed during the current study are not publicly available due to the fact that individual privacy could be compromised but are available from the corresponding author on reasonable request.

## Abbreviations

mNGS: metagenomic next-generation sequencing
BALF: bronchoalveolar lavage fluid
DM: diabetes mellitus
PCR: polymerase chain reaction
EBV: Human gammaherpesvirus 4
CMV: Human betaherpesvirus 5
DNBs: DNA nanoballs
RCA: rolling circle replication
SMRN: stringently mapped read number
ICU: intensive care unit
